# Use of intraoperative fluoroscopy to reduce post–peroral endoscopic myotomy reflux: a proof-of-concept study

**DOI:** 10.1016/j.igie.2025.02.003

**Published:** 2025-02-07

**Authors:** Erica Loon, Rahul Karna, Amir Sultan Seid, Mohammad Bilal, Nabeel Azeem

**Affiliations:** 1 Department of Internal Medicine, University of Minnesota Medical Center, Minneapolis, Minnesota, USA; 2 Division of Gastroenterology and Hepatology, University of Minnesota Medical Center, Minneapolis, Minnesota, USA; 3 Department of Gastroenterology, Minneapolis Veterans Affairs Health Care System, Minneapolis, Minnesota, USA

## Abstract

**Background and Aims:**

Post–peroral endoscopic myotomy (POEM) gastroesophageal reflux is a common adverse event. Intraoperative fluoroscopy (IOF) can help identify the gastroesophageal junction (GEJ) during submucosal tunneling and evaluate the extent of myotomy into the stomach during POEM. In this study, we investigated the use of IOF to predict and prevent post-POEM GERD (PPG).

**Methods:**

This was a retrospective review of all patients who underwent POEM with IOF at our institution. A blinded gastroenterologist measured the fluoroscopic angle (FA) between the endoscope tip at the GEJ, before submucosal tunneling, and at the distal extent of the submucosal tunnel into the cardia. The FA was compared in patients with and without PPG at 3 and 12 months.

**Results:**

Sixty-seven patients were included. The median FA was wider in patients on a proton pump inhibitor at 3 months (10.30 vs –1.35 degrees, *P* = .28) and 12 months (6.20 vs –1.05 degrees, *P* = .46) and in patients with presence of heartburn symptoms at 3 months (6.20 vs 2.35 degrees, *P* = .49) and 12 months (15.10 vs 0.15 degrees, *P* = .16).

**Conclusions:**

Our study suggests that IOF could be used to tailor the myotomy to preserve sling fibers and in turn reduce PPG. Although our findings did not show statistical significance, the trend toward increased PPG in patients with a wider FA warrants a prospective controlled study to further test this hypothesis.

Peroral endoscopic myotomy (POEM) is the primary treatment for achalasia because of its clinical efficacy (∼95%) and minimal adverse events.^1^^,^^2^ However, post-POEM GERD (PPG) is a persistent issue. GERD, defined subjectively as heartburn symptoms and objectively as esophagitis on endoscopy or acid exposure on pH monitoring, is reported in 40% to 60% of cases after POEM.^3^^,^^4^

PPG has been attributed to gastric myotomy >2.5 cm and disruption of oblique and/or sling fibers.^4^ Several studies have proposed technical modifications to address PPG including shorter myotomy, oblique fiber preservation, and reinforcement of diaphragmatic hiatuses with minimal success.^5^, ^6^, ^7^ Intraoperative fluoroscopy (IOF) can be used to identify the gastroesophageal junction (GEJ) during submucosal tunneling and guide the extent of myotomy into the stomach.^8^

In this retrospective study, we investigated the relationship between fluoroscopic angle (FA) during myotomy and PPG. We hypothesized that a wider FA between the GEJ and distal extent of the myotomy would lead to disruption of the angle of His and increased risk for PPG.

## Methods

This was a retrospective review of all patients who underwent POEM using IOF at our institution between May 2019 and October 2023. Patients with unavailable fluoroscopic images and those with history of upper GI surgery (except Heller myotomy or Dor and/or Nissen fundoplication) were excluded.

### IOF and POEM details

All POEMs were performed by a single operator (N.A.) with experience in submucosal endoscopy using the posterior approach. With the patient in a supine position, a diagnostic upper endoscopy was performed. The gastroscope was positioned at the GEJ in a neutral scope position, and a fluoroscopic image was captured with the c-arm centered over the patient to visualize the distal gastroscope and gastric bubble. The POEM procedure then commenced. For type I achalasia, type II achalasia, and esophagogastric junction outflow obstruction, a 3- to 8-cm myotomy was performed in the esophageal body and extended 2 cm into the cardia. Myotomy length was tailored per the spastic segment in type III achalasia or other spastic esophageal disorders.

During submucosal tunneling, fluoroscopy was used to confirm when the scope reached 2 to 3 cm into the cardia, using the initial image as reference. The c-arm did not move during this time. Once confirmed, a fluoroscopic image was saved showing the gastroscope tip at the maximal extent of the tunnel. During any use of fluoroscopy, the gastroscope handle was left on the table to allow the endoscopist to step away. The rest of the myotomy was then completed. After the procedure, all patients were prescribed a proton pump inhibitor (PPI) twice daily for 1 month.

### Definitions and outcomes

Patients underwent clinic follow-up at 1 and 3 months and endoscopy at 12 months. Patients were asked at their 1-month follow-up to stop their antacids to monitor for PPG. PPG was defined as PPI use or heartburn symptoms post-POEM. Heartburn was defined as an occasional or daily burning sensation in the chest that was worse after eating or at night. Regurgitation and dysphagia were not included in screening for PPG because these are common symptoms of achalasia. Primary outcome was comparison of the FA in patients with and without PPG at 3 and 12 months. The secondary objective was a comparison of the FA in patients with and without the presence of esophagitis on endoscopy (Los Angeles classification) at 12 months. Clinical success of POEM was defined as a postoperative Eckardt score ≤3.

### FA measurement

**Figure 1:**
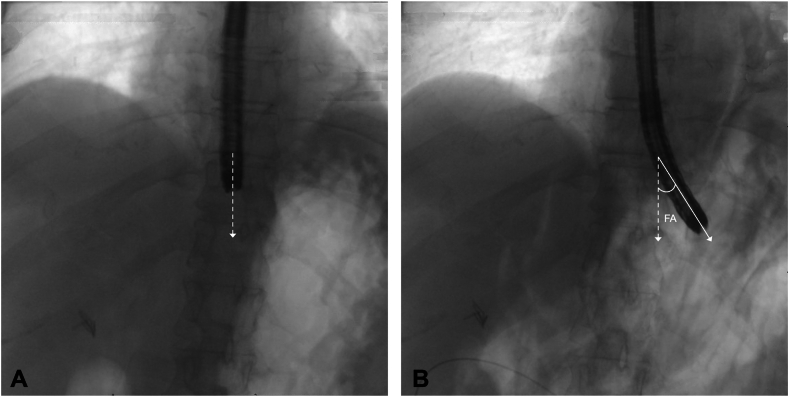
**A,** Fluoroscopic image of the tip of the endoscope at the gastroesophageal junction before submucosal tunneling, with the *dashed line* identifying the initial neutral position at the gastroesophageal junction. **B,** Fluoroscopic image of the tip of the endoscope at the distal extent of the submucosal tunnel into the cardia, with the *solid line* identifying the direction of the myotomy, and formation of the fluoroscopic angle. *FA*, Fluoroscopic angle.

The FA was calculated by a blinded gastroenterologist using the saved fluoroscopic images. The initial neutral position at the GEJ was used as the reference (Fig. 1A). The angle between the endoscope tip at the GEJ, before submucosal tunneling, and the tip at the distal extent of the submucosal tunnel into the cardia was measured (Fig. 1B).

### Statistical analysis

Categorical variables are reported as number and percentages. Continuous variables are reported as median and interquartile range (IQR) or mean and standard deviation, and comparisons were made using the Mann-Whitney U test or 2-sample t-tests for bivariant normally distributed continuous variables, respectively. Statistical analysis was performed with SPSS version 23.0 (IBM Corp, Armonk, New York, USA). A *P* < .05 was used as the level of significance.

## Results

### Demographic details

Sixty-seven patients were included (Table 1). The median age was 65.1 years (IQR, 45.5-75.7), and 52.2% were men. Most patients had type II achalasia (67.2%). The mean lengths of the myotomies in the esophagus and cardia were 8.3 (3.94 cm) and 2.2 (0.49) cm, respectively.

**Table 1:** Patient demographics (n = 67)

Characteristics	Values
Body mass index, kg/m^2^	27.7 (24.5-31.9)
Overweight	21
Class I	14
Class II	6
Class III	5
Race	
White	60
Black	3
Native American or Alaskan Native	1
Asian or Pacific Islander	2
Other	1
Active smoker	8
Active alcohol use	33
History of hiatal hernia	24
History of gastroparesis	5
Type of achalasia	
Type I	9
Type II	45
Type III	10
EGJOO	1
EGJOO and jackhammer	1
Sigmoid	2
Nonsurgical previous achalasia therapies	
Botox injection	11
Pneumatic dilation	7
Nonpneumatic dilation	17
Surgical previous achalasia therapies	
Heller myotomy and Dor fundoplication	5
Heller myotomy, Dor fundoplication, and Nissen fundoplication	1
Heller myotomy alone	1
Presence of heartburn symptoms	
Pre-POEM	61/67
Post-POEM (3 mo)	25/53
Post-POEM (12 mo)	19/29
Proton pump inhibitor use	
Pre-POEM	41/67
Post-POEM (3 mo)	25/53
Post-POEM (12 mo)	13/29
Eckardt Score	
Pre-POEM	6 (5-8)
Post-POEM (3 mo)	0 (0-1)

Values are median (IQR), n, or n/N.

*EGJOO*, Esophagogastric junction outflow obstruction; *POEM*, peroral endoscopic myotomy.

### Procedure outcomes

One month after POEM, 75.5% of patients (40/53) were able to be weaned off their antacids. The 3-month follow-up rate was 79.1% (53/67). The 12-month follow-up rate was 43.3% (29/67) with a 30% endoscopy rate (20/67), because most patients had yet to reach 12 months post-POEM. Of the 38 patients who did not have a clinic follow-up at 12 months, 21 (55.3%) had not reached that time point for their follow-up and 15 (39.5%) were lost to follow-up at 12 months. Similarly, of the 47 patients who did not have data for the 12-month endoscopy, 21 (44.7%) had not made it to that appointment time yet and 24 (51.1%) were lost to follow-up for that appointment. Clinical success at 3 months was 94.2% (50/53). At the 3-month follow-up, 47.2% of patients (25/53) reported using PPIs and 9.4% (5/53) reported using histamine 2 receptor antagonists. At 12 months, 44.8% (13/29) and 20.6% (6/29) reported PPI and histamine 2 receptor antagonist use, respectively. Heartburn was reported by 47.2% of patients (25/53) at 3 months and 65.5% (19/29) at 12 months. At 12 months, 50.0% (10/20) had esophagitis (Los Angeles grade A in 7, B in 2, and C in 1) on endoscopy, with one-half not on PPIs. One patient required fundoplication for PPG.

**Figure 2:**
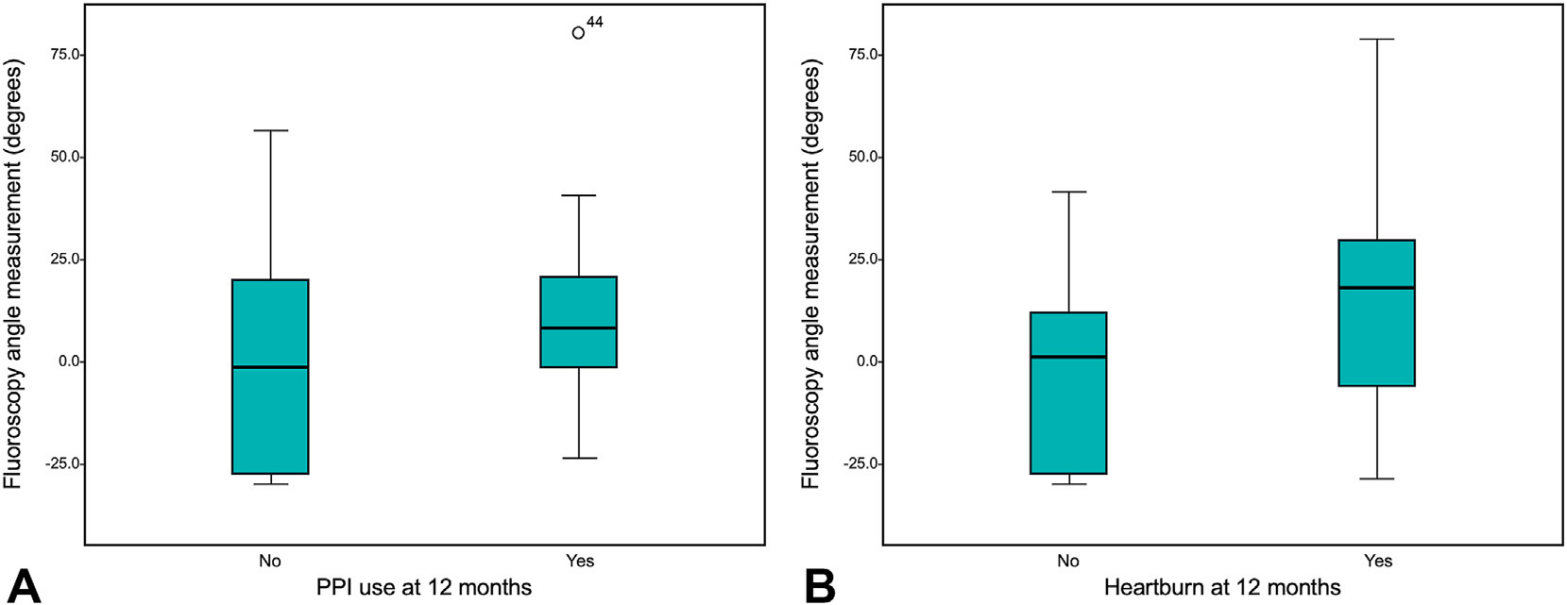
**A,** The median (*center black line* in the box), interquartile range (extent of the boxes), and the minimum/maximum (*whiskers*) fluoroscopic angle in patients on PPIs at 12 months after peroral endoscopic myotomy (POEM). **B,** The median (*center black line* in the box), interquartile range (extent of the boxes), and the minimum/maximum (*whiskers*) fluoroscopic angle in patients with heartburn symptoms at 12 months after POEM. *PPI*, Proton pump inhibitor.

There was a trend toward a higher median FA in patients on PPIs compared with patients not on PPIs at 3 months (10.30 vs –1.35 degrees, *P* = .28) and 12 months (6.20 vs –1.05 degrees, *P* = .46) (Fig. 2A). Similarly, a trend of higher median FAs was observed in patients with heartburn compared with those without heartburn at 3 months (6.20 vs 2.35 degrees, *P* = .49) and 12 months (15.10 vs 0.15 degrees, *P* = .16) (Fig. 2B).

### Adverse events

Five intraprocedural adverse events occurred: bleeding in 3 patients, managed intraoperatively, and aspiration during intubation in 2 patients. Seven postprocedure adverse events occurred: 2 cases of self-resolving chest spasms, 1 patient with a history of Crohn’s disease and Heller myotomy with Dor fundoplication developing idiopathic dehiscence of the mucostomy site and requiring esophageal stent placement, 1 self-resolving small pneumothorax, 1 self-resolving pleural effusion, 1 case of cholecystitis requiring cholecystectomy, and 1 fatal case of respiratory failure in a patient with pre-existing pulmonary disease.

## Discussion

The POEM clinical success rate (94.2%) was similar to previous trials.^2^ Subjective PPG was observed in nearly 46% and 57% of patients at 3 and 12 months, respectively, similar to prior reports (40%-60%).^3^^,^^4^ Patients with PPG at 3 and 12 months post-POEM had a wider FA compared with patients without PPG, although these findings did not reach statistical significance.

The angle of His, located at 8 o’clock at the level of the lower esophageal sphincter during a posterior POEM, acts as an antireflux valve.^9^ Tunneling between 5 and 6 o’clock is suggested to preserve the angle, but reliable methods to ensure endoscopists are at this position at the GEJ within the tunnel are lacking. We propose using the FA to avoid disrupting the angle of His and preventing PPG. A wider FA, meaning a myotomy that moves closer to the fundus rather than the lesser curvature in relation to the GEJ, may increase PPG risk through disruption of the oblique and/or sling muscle fibers, causing angle collapse and backflow pressure on the distal esophagus.^10^ Previous studies found an association between fiber preservation and reduced esophagitis.^10^^,^^11^

A recent randomized trial comparing fiber preservation with conventional myotomy showed no difference in PPG at 1 year.^5^ However, most oblique fibers were likely preserved in both cohorts, because the angle of myotomy did not change until well into the cardia.^12^ Our study contributes to the literature by directly measuring the angle of myotomy, reducing subjectivity in assessing potential disruption of the angle of His and accounting for the outliers (wider angles).

IOF during POEM requires easy access to fluoroscopy, which may not be available at all endoscopy units. An alternative for assessing the extent of the submucosal tunnel is the double-scope method, which offers direct visualization of the GEJ and cardia with real-time feedback. However, this method is cumbersome, requiring 2 endoscope towers and often a second endoscopist.^13^

Our study had several limitations. This was a retrospective, single-operator, single-center study with a limited patient sample. Low 12-month follow-up and endoscopy rates limited the power of our study and our use of objective data to define PPG. Thirteen patients were unable to wean off their antacids after 1 month after POEM because of heartburn symptoms (5/13), persistent achalasia (4/13), or comorbid conditions requiring an antacid (4/13), which may introduce confounding variability. Additionally, confounding variables for PPI use and heartburn symptoms (hereditary risk, body habitus, lifestyle, and comorbid conditions) were not evaluated.^14^^,^^15^ Inclusion of post-Heller myotomy and fundoplication patients may also have affected PPG rates, although their impact on FA is uncertain.^16^

Although our findings did not meet statistical significance, the trend toward increased PPG in patients with a wider FA suggests that IOF could be used to tailor the myotomy to preserve oblique and/or sling fibers and reduce PPG. Future prospective studies with a larger cohort are needed to further test this hypothesis.

## Patient Consent

Informed consent was obtained from the patients for the publication of their information and imaging.

## Disclosure

The following authors disclosed financial relationships: M. Bilal: Consultant for Boston Scientific; speaker for Cook Endoscopy. N. Azeem: Consultant for Boston Scientific. All other authors disclosed no financial relationships.
